# The impact of HAART initiation timing on HIV-TB co-infected patients, a retrospective cohort study

**DOI:** 10.1186/1471-2334-14-304

**Published:** 2014-06-04

**Authors:** Chin-Hui Yang, Kuan-Jung Chen, Jih-Jin Tsai, Yu-Hui Lin, Shu-Hsing Cheng, Kwei-Feng Wang, Hung-Yi Chiou

**Affiliations:** 1 Centers for Disease Control, Ministry of Health and Welfare, 5F, No.6, Linsen S. Rd., Taipei City 100, Taiwan; 2 Ph. D. program in School of Public Health, Taipei Medical University, No. 250, 469 Wu-Hsing Street, Taipei, Taiwan; 3 Department of Chest Medicine, RenAi branch of Taipei City Hospital, No. 10, Section 4, Ran-Ai Road, Taipei, Taiwan; 4 School of Medicine, College of Medicine, Kaohsiung Medical University, No.100, Shih-Chuan 1st Road, Kaohsiung, Taiwan; 5 Division of Infectious Diseases, Department of Internal Medicine, Kaohsiung Medical University Hospital, , No.100, Tzyou 1st Road, Kaohsiung, Taiwan; 6 Tropical Medicine Center, Kaohsiung Medical University Hospital, School of Medicine, College of Medicine, Kaohsiung Medical University, No.100, Tzyou 1st Road, Kaohsiung, Taiwan; 7 Section of Infectious Diseases, Department of Internal Medicine, Taichung Veterans General Hospital, No.1650, Sec. 4, Taiwan Boulevard, Taichung, Taiwan; 8 Section of Infectious Diseases, Department of Internal Medicine, Taoyuan General Hospital, No. 1492, Chung-Shan Road, Taoyuan, Taiwan; 9 School of Public Health, Taipei Medical University, No. 250, Wu-Hsing Street, Taipei, Taiwan

**Keywords:** Tuberculosis, HIV, Mortality, HAART

## Abstract

**Background:**

Optimal timing for initiating highly active antiretroviral therapy (HAART) in HIV-TB coinfected patients is challenging for clinicians. We aim to evaluate the impact of different timing of HAART initiation on TB outcome of HIV-infected adults in Taiwan.

**Methods:**

A population-based retrospective cohort study was conducted through linking the HIV and TB registries of Taiwan Centers for Disease Control (CDC) during 1997 to 2006. Clinical data of HIV-TB co-infected patients, including the presence of immune reconstitution inflammatory syndrome (IRIS), was collected through medical records review. The outcome of interest was all-cause mortality within 1 year following TB diagnosis. The Cox proportional hazard model was used to explore the probability of death and IRIS after TB diagnosis by adjusting for confounding factors and factors of interest. The probability of survival and TB IRIS were calculated by the Kaplan-Meier method and compared between different HAART initiation timing groups by the log-rank test.

**Results:**

There were 229 HIV-TB co-infected patients included for analysis and 60 cases (26.2%) died within one year. Besides decreasing age and increasing CD4 lymphocyte count, having started HAART during TB treatment was significantly associated with better survival (adjusted Hazard Ratio was 0.11, 95% CI 0.06–0.21). As to the timing of HAART initiation, there was only non-significant benefit on survival among cases initiating HAART within 15 days, at 16–30 days and at 31–60 days of TB treatment than initiating after 60 days. Cases with HAART initiated after 30 days had lower risk in developing IRIS than cases with HAART initiated earlier. Cases with IRIS had significantly higher rate of re-hospitalization (49% vs. 4%, p < 0.001) and prolonged hospitalization (28 days vs. 18.5 days, p < 0.01).

**Conclusion:**

The present study found that starting HAART during TB treatment is associated with better one-year survival, although earlier initiation within 60 days of TB treatment did not show statistical differences in survival than later initiation. Initiation of HAART within 30 days appeared to increase the risk of IRIS. Deferring HAART to 31–60 days of TB treatment might be optimal after considering the risks and benefits.

## Background

Among the 1.3 million people who died from tuberculosis (TB) in 2012, one-quarter were also HIV-infected [1]. Although highly active antiretroviral therapy (HAART) can reduce the mortality of HIV-TB co-infected patients, HIV-infected individuals still had a higher TB mortality rate than HIV-uninfected individuals [2-5].

Various aspects of therapy, such as drug interactions, overlapping toxicity and the risk of immune reconstitution inflammatory syndrome (IRIS), make the treatment of TB complex in HIV-infected persons. Observational studies found that deferral of HAART in TB treatments are associated with higher mortality [6,7]. Nevertheless, early initiation of HAART during TB treatment is strongly associated with the occurrence of IRIS [8,9]. Although deaths resulting directly from TB IRIS appear to be limited, the development of IRIS usually results in higher hospitalization rate, interruption of TB treatment and longer time to resolution [10,11].

Results from randomized controlled trials (RCTs) provide evidences regarding the timing of HAART initiation and treatment outcome among HIV-TB co-infected patients. The SAPiT trial in South Africa and ACTG 5221 STRIDE trial in Africa and North America both demonstrated that early initiation of HAART (within 2–4 weeks after TB treatment) can reduce AIDS events and deaths in persons with CD4+ lymphocyte counts ≤ 50 cells/mm^3^, though no significant benefits were observed in patients with higher CD4+ lymphocyte counts [12,13]. CAMELIA trial in Cambodia showed that early initiation of HAART can reduce mortality in patients with CD4+ lymphocyte counts ≤ 200 cells/mm^3^[14]. TIME study in Thailand, on the other hand, found that early initiation of HAART was not associated with survival advantages [15]. Because the study design of RCTs usually excluded patients with abnormal liver function, the results might not be generalizable to areas with high HBV and HCV prevalence such as Taiwan [16]. In addition, because the majority of the participants in the trials were from resource constrained countries, the results might not be applicable to countries with better resources.

Taiwan, with moderate TB burden (2011 prevalence was 54.5 per 100,000 population ) and low HIV prevalence (0.16% in 2011), has good health infrastructure and public health system to implement national HIV and TB programs [17,18]. There were 12,634 new TB cases in 2011 and around 22,000 persons living with HIV by the end of 2011 [19].

We conducted a retrospective cohort study of HIV-TB co-infected patients from 1997 to 2006 through medical records review. Clinical data collected included the date of HAART initiation, presence of IRIS and one-year survival, stratified by CD4+ lymphocyte counts of 50 cells /mm^3^. Our aim was to understand the TB outcome of HIV-infected adults under routine programmatic conditions in Taiwan and hope to contribute to the understanding of the optimal timing to initiate HAART in co-infected patients.

## Methods

### Study settings

Both HIV infection and tuberculosis were mandatorily reportable in Taiwan since 1984. Cases of HIV infection detected by ELISA must be confirmed by Western blot. HIV/AIDS infected individuals are provided with free medical care by the government of Taiwan, including HAART, which was introduced in 1997 [20]. Computerized National Tuberculosis Registry was established in 1994 and physicians are required to report all suspected and confirmed TB cases within 7 days [21]. There are no parallel reporting systems for laboratories or pharmacies.

### Data collection

We linked all data prior to 2007 in the HIV and TB registries of Taiwan CDC to identify HIV-TB co-infected patients. Patients with HIV diagnosed before TB diagnosis or within 5 months after TB diagnosis were enrolled. To assess the effect of HAART on the survival of patients with TB and to avoid biases resulted from antiretroviral use, we only included patients with TB diagnosed after 1997 who were not on HAART when TB was diagnosed for analysis. All medical and microbiologic records were reviewed by physicians to obtain clinical information which included date of diagnosis; type of TB (pulmonary or extra-pulmonary); antiretroviral drugs used before and after the diagnosis of TB; and CD4+ lymphocyte count and HIV viral load closest to the date of TB diagnosis. For patients with significant adverse effects from drugs or IRIS during anti-TB treatment, clinical records were reviewed.

### Definitions

We only included bacteriologically confirmed TB cases, which was defined as: (1) a positive smear of acid-fast bacilli (AFB) without culture result and disease clinically compatible with TB infection; (2) a successful culture of *Mycobacterium tuberculosis* (MTB) from pulmonary or extra-pulmonary specimens. TB was divided into pulmonary (disease localized to the lungs only) or extra-pulmonary (disease anywhere outside the lungs).

HAART was defined as combination therapy of at least 3 antiretroviral drugs that included at least a protease inhibitor or non-nucleoside reverse transcriptase inhibitor. The most common HAART regimen for HIV-TB co-infected patients consisted of stavudine or zidovudine, lamivudine and efavirenz. If patients experienced adverse effects, they were shifted to a protease-inhibitor-based ART regimen and rifampin in their TB-treatment regimen was replaced with rifabutin [22]. Both anti-TB and HAART were self-administered by patients.

Enrolled patients were divided into 5 groups: patients who did not receive HAART during TB treatment; patients whose HAART was initiated within 14 days; between 15–30 days; between 31–60 days; and over 60 days from the date of starting TB treatment. IRIS was defined as deterioration in either clinical status or laboratory findings following the initiation of antiretroviral therapy in the absence of other causes.

The outcome of interest studied was all-cause mortality within 1 year following TB diagnosis and treatment. Death at 1 year post-TB diagnosis was determined from medical records and verified using Taiwan’s National Death Registry. Deaths were determined to be TB-related if two or more of the following conditions were met: TB was listed as a cause of death in the pathological examination report; TB was identified in discharge summaries as a probable cause of death; or, patient had a positive AFB smear or culture for *Mycobacterium tuberculosis* from any specimens taken within 6 weeks prior to death.

### Statistical analysis

Categorical variables were described by their absolute counts and percentages. Numerical variables were described by median with interquartile range at 25^th^ and 75^th^ (IQR). χ^2^ tests and Student’s t-test were used to compare categorical and continuous variables, respectively. The Cox proportional hazard model was used to explore the probability of death and IRIS after TB diagnosis by adjusting for confounding factors and factors of interest. Factor which had p value of less than 0.15 by univariate analysis and factors of interest were included into the multivariate analysis. The results were expressed as hazard ratios (HRs) with 95% confidence intervals (CI). The probability of survival and TB IRIS were calculated by the Kaplan-Meier method and compared between different HAART initiation timing groups by the log-rank test. All comparisons were two-tailed with p values < 0.05 considered to be statistically significant. Statistical analysis was performed using SAS, version 9.3, software (SAS Institute, Cary, North Carolina, USA).

### Ethics

The study was approved by the Centers for Disease Control, Ministry of Health and Welfare, Executive Yuan, Taiwan. To preserve patient confidentiality, only the author or physicians working in the designated hospitals were responsible for data extraction from patients’ medical records. Moreover, no personal identifiers were used on the data collection form.

## Results

**Figure 1:**
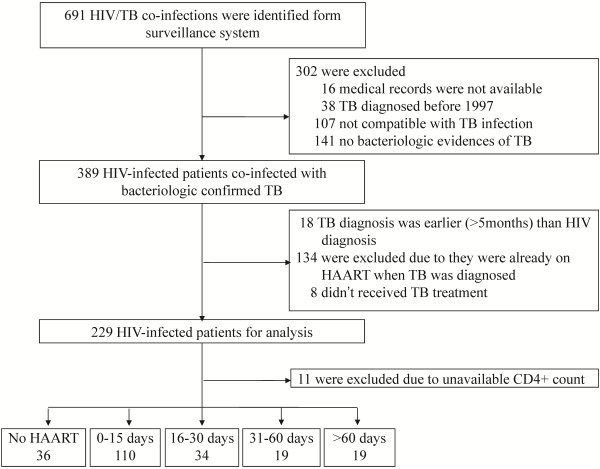
Flow chart showing HIV-TB co-infected individuals recruited for analysis.

Among the 13,013 reported HIV-infected persons in Taiwan by the end of 2006, there were 389 patients (3.0%) who had at least 1 episode of bacteriologically confirmed TB occurring after 1997. Excluding 134 patients who were already on HAART when TB was diagnosed, a total of 229 cases met the inclusion criteria for analysis (Figure 1). The demographic data is shown in Table 1. The median CD4+ lymphocyte count at TB diagnosis was low and did not vary with time. The 11 cases without CD4+ lymphocyte count data had the highest mortality rate and 9 of them did not initiate HAART. Other than being significantly older, these patients showed no other demographic differences from the other patients. There were 60 patients (26.2%) who died within one year; 27 deaths (45.0%) occurred within 60 days after TB diagnosis. Only older age and patients without HAART during TB treatment were associated with death; the p value was 0.002 and <0.001 respectively.

**Table 1:** Characteristics of 229 HIV-infected patients co-infected with tuberculosis

	**Died (*n*** **= 60, 26.2%)**	**Survived (n = 169)**	**Total (n = 229)**
TB diagnosis year			
1997–2000	12 (20.3%)	47 (79.7%)	59 {25.8%}
2001–2003	19 (29.2%)	46 (70.8%)	65 {28.4%}
2004–2006	29 (27.6%)	76 (72.4%)	105 {45.9%}
Age (yrs) at first TB diagnosis, median [IQR]^$^	42.1 [33.3-53.5]	36.8 [32.4-43.6]	37.5 [32.5-47.1]
Male	55 (25.6%)	160 (74.4%)	215 {93.9%}
Female	5 (35.7%)	9 (64.3%)	14 {6.1%}
HIV transmission category			
MSM	18 (20.0%)	72 (80.0%)	90 {39.3%}
Heterosexual contacts	33 (39.7%)	78 (70.3%)	111 {48.5%}
IDUs	4 (19.1%)	17 (81.0%)	21 {9.2%}
Other/undefined	5 (71.4%)	2 (28.6%)	7 {3.1%}
CD4 count (/mm^3^) at TB diagnosis, median [IQR]	22 [7-85]	43 [20-117]	40 [17-107]
≤50	32 (25.2%)	95 (74.8%)	127 {55.5%}
51–100	9 (25.0%)	27 (75.0%)	36 {15.7%}
101–200	8 (33.3%)	16 (66.7%)	24 {10.5%}
≥200	3 (9.7%)	28 (90.3%)	31 {13.5%}
No data	8 (72.7%)	3 (27.3%)	11 {4.8%}
Localization of tuberculosis			
Pulmonary	22 (26.8%)	60 (73.2%)	82 {35.8%}
PTB+ extra-pulmonary	37 (28.2%)	94 (71.8%)	131 {57.2%}
Extra-pulmonary	1 (6.3%)	15 (93.8%)	20 {7.0%}
Laboratory findings			
Positive AFS smear	46 [76.7%]	127 [75.2%]	173 {75.6%}
Positive MTB culture	49 [81.7%]	139 [82.3%]	188 {82.1%}
HAART during anti-TB treatment^$^			
No	30 (66.7%)	15 (33.3%)	45 {19.7%}
Yes	30 (16.3%)	154 (83.7%)	184 {80.3%}
IRIS*	5 (8.8%)	52 (91.2%)	57 {31.0%}*
HBV (+)^#^	7 [11.7%]	29 [17.2%]	36 {15.7%}
HCV (+)^#^	5 [8.3%]	22 [13.0%]	27 {11.8%}

Data are presented as n (% of the category) unless otherwise indicated. {%} indicated as the proportion of the total reported cases. [%] indicated as the proportion in the subgroup.

*Abbreviations*: *IQR* interquartile range, *PTB* pulmonary tuberculosis. *AFS* acid-fast stain, *MTBMycobacterium tuberculosis*, *MSM* men who had sex with men, *IDUs* injecting drug users, *HAART* highly active antiretroviral therapy, *IRIS* Immune reconstitution inflammatory syndrome, *HBV* hepatitis B virus infection, *HCV* hepatitis C virus infection.

*Only cases started HAART were counted as denominator.

#Only 156 and 144 patients had available HBV and HCV examination results respectively.

$ p < 0.01.

There were 45 patients who did not receive HAART while being treated for TB. These patients were significantly older, had higher CD4+ lymphocyte count, and higher rate of HCV co-infections. Possible reasons for not starting HAART included: 7 cases had CD4+ lymphocyte count ≥200 cells/mm^3^ (all survived); 3 cases defaulted from TB treatment (all survived); 10 cases died within 15 days after TB diagnosis; 8 cases had other conditions (3 with abnormal liver function because of liver cirrhosis; 2 with cancer; 1 committed suicide; 1 had severe sepsis; and 1 had personal reasons; all of them died). There was 1 case that the reason for not starting HAART remained unknown and died at 54 days after TB treatment. There were 4 patients whose HIV diagnoses were delayed for over 1 month after TB diagnosis, and they all died. As to the other 12 cases, their medical records did not mention HIV infection during the diagnosis and treatment of TB, and 7 of them died.

Further analysis of the 218 patients with CD4+ lymphocyte counts available, the highest mortality rate occurred among the 36 patients who never initiated HAART (Table 2). The next highest mortality rate was observed among the 19 patients whose HAART was delayed for at least 60 days (26.3%), followed by patients in whom HAART began within 15 days (17.3%), in 16–30 days (14.7%) and 31–60 days (0%). There were 39 TB-related deaths (75.0%), and their mortality rate trend was similar to that of all deaths. Cases with HCV co-infection had significantly lower chance to initiate HAART during TB treatment than cases without or had unknown HCV status (p < 0.01); HBV co-infection did not show such findings. One third of the patients developed IRIS after initiation of HAART. The median duration from HAART initiation to the development of IRS was 10 days (range 1–37 days). The incidence of IRIS was significantly higher in patient who started HAART within 30 days compared with those who started after 30 days (36.8% vs. 10.5%, p < 0.05). The mortality rate was lower among patients with IRIS than those without IRIS (8.8% vs. 19.2%, p = 0.08). Five deaths among patients with IRIS all had HAART initiated within 30 days after TB treatment. Patients who developed IRIS had significantly higher rate of re-hospitalization than those without IRIS (49% vs. 4%, p < 0.001). Patients who developed IRIS also had prolonged mean hospitalization duration than those without (28 days vs. 18.5 days, p < 0.01).

**Table 2:** Characteristics of the 218 HIV-TB co-infected patients divided by initiation timing of HAART*

	**HAART initiation timing during anti-TB therapy**				
	**No HAART (n = 36)**	**0–15 days (n = 110)**	**16–30 days (n = 34)**	**31–60 days (n = 19)**	**>60 days (n = 19)**
Age (yrs) at first TB diagnosis, median [IQR]	38.8 [32.0–49.6]	36.8 [31.8–43.6]	35.5 [31.2–46.7]	36.6 [33.7–45.1]	40.4 [29.5–61.3]
Male	31 (86.1%)	107 (97.3%)	33 (97.1%)	18 (94.7%)	17 (89.5%)
Female	5 (13.9%)	3 (2.7%)	1 (2.9%)	1 (5.3%)	2 (10.5%)
CD4 count(/mm^3^) at TB diagnosis, median [IQR]^$^	69 [17–370]	37 [16–87]	34 [14–62]	68 [23–134]	36 [17–94]
≤50	14 (38.9%)	68 (62.4%)	26 (74.3%)	8 (42.15%)	11 (57.9%)
51–100	7 (19.4%)	18 (16.4%)	2 (5.9%)	5 (26.3%)	4 (21.1%)
101–200	5 (13.9%)	11 (10.1%)	3 (8.6%)	4 (21.1%)	1 (5.3%)
≥200	10 (30.8%)	13 (11.9%)	3 (8.6%)	2 (10.5%)	3 (15.8%)
Localization of tuberculosis					
Pulmonary	16 (44.4%)	31 (28.5%)	15 (42.9%)	6 (31.6%)	7 (36.8%)
PTB+ extra-pulmonary	19 (52.8%)	72 (65.5%)	19 (55.9%)	6 (31.6%)	12 (63.2%)
Extra-pulmonary	1 (2.8%)	7 (6.4%)	0	7 (36.8%)	0
Laboratory findings					
Positive AFS smear ± positive MTB Culture	27 (75.0%)	85 (78.0%)	29 (82.9%)	13 (68.4%)	12 (63.2%)
Positive MTB culture only	9(25.0%)	25(22.7%)	5(14.7%)	6(31.6%)	7(36.8%)
HBV(+)^#$^	6(16.7%)	22(20.2%)	1(2.9%)	4(21.1%)	3(15.8%)
HCV(+)^#$^	9(25.0%)	11(10.1%)	3(8.6%)	1(5.3%)	1(5.3%)
IRIS^$^	–	41(37.3%)	12(35.3%)	2(10.5%)	2(10.5%)
Death^$^	23(63.9%)	19(17.3%)	5(14.7%)	0	5(26.3%)
TB-related death^$^	19(52.8%)	15(13.6%)	2(5.9%)	0	3(15.8%)

Data are presented as n (% of the category) unless otherwise indicated.

*Abbreviations*: *IQR* interquartile range, *PTB* pulmonary tuberculosis. *AFS* acid-fast stain, *MTBMycobacterium tuberculosis*, *IRIS* Immune reconstitution inflammatory syndrome, *HBV* hepatitis B virus infection, *HCV* hepatitis C virus infection.

*Only included cases with available CD4+ lymphocyte count.

#Only 153 and 141 patients had available HBV and HCV examination results respectively.

$ p <0.01.

**Figure 2:**
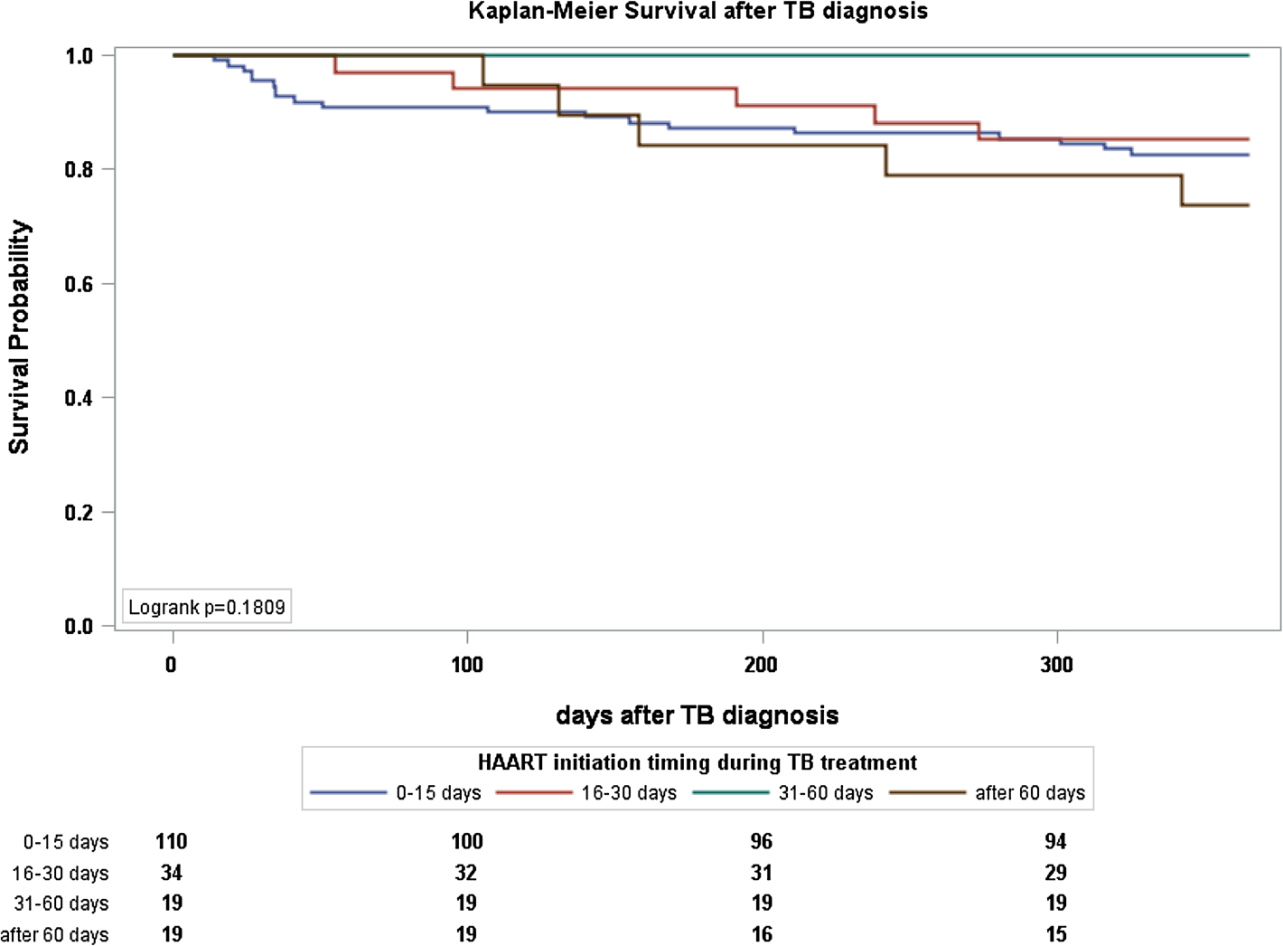
**Kaplan-Meier plot of survival after TB diagnosis stratified by the timing of HAART initiation.** Use HAART at 31–60 days as reference, the *p* value by log-rank test was 0.03 for HAART after 60 days; *p* value by log-rank test was both >0.05 for HAART at 0–15 days and at 16–30 days.

**Figure 3:**
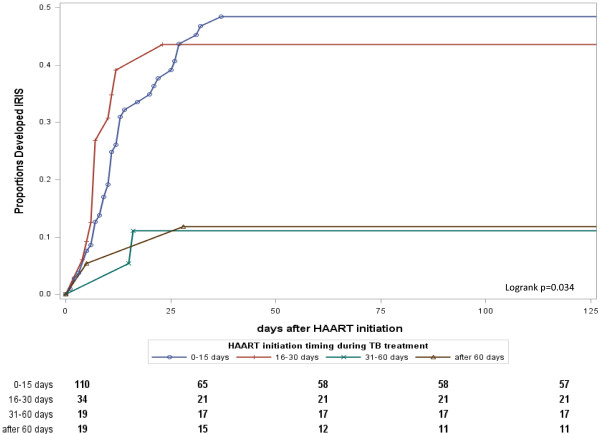
**Kaplan-Meier plot of TB-IRIS occurrence after HAART stratified by the timing of HAART initiation.** Use HAART at 31–60 days as reference, *p* value by log-rank test was 0.02 and 0.08 for HAART at 0–15 days and at 16–30 days; *p* value by log-rank test was 0.85 for HAART after 60 days. The *p* value by log-rank test between HAART after 60 days and 0–15 days was 0.02.

Multivariate analysis of factors associated with all-cause mortality among cases with different timing of HAART initiation was conducted (see Table 3). Increasing CD4+ lymphocyte counts and decreasing age were associated with favorable outcomes. Having started HAART during TB treatment was associated with significantly better survival and the aHR was 0.11 (95% CI 0.06–0.21); stratifying the cases into CD4+ lymphocyte counts >50 cells/mm^3^ and ≤50 cells/mm^3^ showed similar results, with aHR of 0.08 (95% CI 0.03-0.17) and 0.12 (95% CI 0.04-0.37), respectively (data not shown). Among patients with CD4+ lymphocyte counts ≤50 cells/mm^3^, no matter when HAART was started, even within 15 days, there was a survival benefit compared to those who never started HAART (see Additional file 1). Use HAART initiated after 60 days as reference, there were no statistical differences in survival compared to patients whose HAART was initiated at 0–15 days and 16–30 days. Further stratify with CD4+ lymphocyte counts 50 cells/mm^3^ showed the same results (see Additional file 1). The cumulative survival probabilities after TB diagnosis of the 4 groups with different HAART initiation timing is shown in Figure 2 (p = 0.18, log-rank test). Cases initiated HAART after 30 days had significantly lower risk in developing IRIS than those who had HAART initiated earlier; the HR was 4.10 (95% CI 1.48–11.33, data not shown). Multivariate analysis of factors associated with IRIS occurrence among cases with different timing of HAART initiation is shown in Table 4 and Figure 3.

**Table 3:** The relationship of mortality and initiation timing of HAART among HIV-TB co-infected patients*

	**Total (n = 218)**	**Death (N = 52, 24%)**	**Hazard ratio (95% CI)**	**Adjusted HR**^**$ **^**(95% CI)**	**Adjusted HR**^**# **^**(95% CI)**
Age at TB diagnosis (per 5-year increase)			1.14 (1.03-1.27)	1.12 (1.01-1.25)	1.13 (0.97-1.32)
CD4 count at TB diagnosis (per 50-cell increase)			0.94 (0.83-1.06)	0.81 (0.71-0.92)	0.79 (0.60-1.05)
IRIS	57	5 (8.8%)	0.26 (0.10-0.65)	0.36 (0.14-0.97)	0.36 (0.13-0.95)
HAART initiation timing during anti-TB therapy					
No HAART	36	23 (63.9%)	1	1	
0–15 days	110	19 (17.3%)	0.17 (0.09-0.31)	0.14 (0.07-0.27)	0.97 (0.34-2.70)
16–30 days	34	5 (14.7%)	0.14 (0.05-0.36)	0.10 (0.04-0.28)	0.69 (0.19-2.49)
31–60 days	19	0 (0%)	–	–	–
>60 days	19	5 (26.3%)	0.25 (0.10-0.67)	0.14 (0.05-0.39)	1

*Abbreviations*: *HAART* highly active antiretroviral therapy, *IRIS* Immune reconstitution inflammatory syndrome.

*Only enrolled cases with available CD4+ lymphocyte count. HBV co-infection, HCV co-infection and TB location were analyzed initially but the p value was greater than 0.15 and was not included for multivariate analysis and not shown in the table.

$ Adjusted for age at TB diagnosis, CD4 + lymphocyte count, IRIS and HAART initiation timing (use no HAART as reference).

#Excluded cases who did not start HAART during TB treatment and adjusted for age at TB diagnosis, CD4+ lymphocyte count, IRIS and HAART initiation timing (use after 60 day as reference).

**Table 4:** The relationship of IRIS occurrence and initiation timing of HAART among HIV-TB co-infected patients*

	**Total (n = 182)**	**IRIS (N = 57, 25%)**	**Hazard ratio (95% CI)**	**Adjusted HR**^**# **^**(95% CI)**	**Adjusted HR**^**$ **^**(95% CI)**
Age at TB diagnosis (per 5-year increase)			0.99 (0.97-1.01)	0.99 (0.97-1.02)	0.99 (0.97-1.02)
CD4 count at TB diagnosis (per 50-cell increase)			0.95 (0.81-1.11)	0.97 (0.83-1.13)	0.97 (0.83-1.13)
HAART initiation timing during anti-TB therapy					
0–15 days	110	41 (71.9%)	1	1	4.24 (1.02-19.5)
16–30 days	34	12 (21.1%)	0.98 (0.51-1.86)	0.96 (0.50-1.84)	4.08 (0.91-18.3)
31–60 days	19	2 (3.5%)	0.23 (0.06-0.96)	0.24 (0.06-0.98)	1
>60 days	19	2 (3.5%)	0.26 (0.06-1.06)	0.27 (0.07-1.12)	1.14 (0.16-8.11)

*Abbreviations:HAART* highly active antiretroviral therapy, *IRIS* Immune reconstitution inflammatory syndrome.

*Only enrolled cases with available CD4+ lymphocyte count and start HAART during TB treatment. HBV co-infection, HCV co-infection and TB location were analyzed initially but the p-value was greater than 0.15 and was not included for multivariate analysis and not shown in the table.

#Adjusted for age at TB diagnosis, CD4+ lymphocyte count and HAART initiation timing (use initiation timing at 0–15 days as reference).

^$^Adjusted for age at TB diagnosis, CD4+ lymphocyte count and HAART initiation timing (use initiation timing at 31–60 days as reference).

## Discussion

Our study showed that patients who had HAART initiated during anti-TB treatment had better one-year survival than those who did not. The benefit was significant for both patients with CD4+ lymphocyte counts >50 cells/mm^3^ and ≤50 cells/mm^3^. There were no statistical differences in one-year survival between patients who had HAART initiated within 15–60 days of TB treatment and those who had delayed HAART initiation to after 60 days. Patients with HAART initiated after 30 days of TB treatment had lower risk of IRIS than those who had HAART initiated within 30 days.

HAART during anti-TB treatment was the most important determinant of one-year survival among HIV-TB co-infected patients, especially in patients with CD4+ lymphocyte counts ≤50 cells/mm^3^. HAART can improve the patients’ immune status and avoid excess deaths because of other opportunistic infections [23,24]. Our study found one third of the deaths were not TB-related. There were 4 patients who died of *Pneumocystis jiroveci* pneumonia, 2 hepatic failures, 2 sepsis, 2 diabetes mellitus with renal failure, 2 hematologic cancers and 1 suicide. The proportion of non-TB deaths was higher among patients without HAART, compared with those who started HAART (11.1% vs. 4.9%, p = 0.15), though the difference is not statistically significant. Unfortunately, we found that some patients missed the chance to start HAART early because of unknown HIV status during anti-TB treatment. There were 16 cases that did not disclose their HIV status and the attending physicians did not recognize or had delayed recognition of the patient’s HIV status during TB treatment. WHO recommended offering HIV testing to all TB patients. The estimated rate of HIV testing reached 46% of notified TB cases in 2012 globally, but implementation in the Western Pacific region is still low, reaching 34% only [1]. HIV testing rate among TB patients is only 28.8% in Taiwan [25]. Furthermore, although HAART is freely available in Taiwan, there are some HIV-infected patients who do not link into HIV-related medical care [26]. The barrier between HIV-TB co-infected patients and medical services is also reported in other studies [27,28]. In mid-2013, the National TB program launch a project to have every TB patient aged 15–49 years complete HIV test. Integration of HIV and TB services to improve HIV testing among TB patients and overcome obstacles are important in reducing these events.

As to the timing of HAART initiation, in 2009, WHO recommend initiating HAART within 15–60 days after anti-TB treatment [29]. Results from studies were inconsistent. Some found early initiation of HAART within 4 weeks after TB treatment can reduce mortality [12-14], but others found the timing had no significant impact [15,30,31]. The benefit of early HAART (within 30 days) is contingent on increased risk and severity of IRIS accompanied with higher re-hospitalization rate and longer TB treatment duration. We have similar observations with previous studies [8,10,32]. In addition, we found several cases had prolonged hospitalization because of persistent positive sputum smear. Although we cannot demonstrate the time to sputum conversion had differed between cases with and without IRIS because of the retrospective study design, prolonged hospitalization hints at this possibility. There were no studies focused on the period of infectiousness following TB IRIS. More research is needed for this important topic, because this may lead to further transmission in the community.

We found that initiating HAART between 31–60 days after TB treatment may be a feasible way to balance the risks and benefits. Our results showed that cases in this group had good survival and the survival probabilities was not different compared with cases that started HAART between 16–30 days, even if the cases had CD4+ lymphocyte counts of ≤50 cells/mm^3^. One retrospective chart review study in Durban had the same observation [30]. This is different from the results of Rwanda’s retrospective cohort analysis, where the authors, using marginal structural models, found that HAART initiation at day 15, instead of later times, was protective against death [33]. We noticed that 11% of our patients had HAART initiation deferred to between 31–60 days after TB treatment. In the SAPiT trial, the study protocol design allowed HAART initiation timing in early-HAART arm to be at the discretion of the physicians. There were 33 cases (15% of original assignment) started HAART after 4 weeks [13]. Although there were no further analyses of the outcome for these patients, it demonstrates that some patients cannot have HAART initiated within 4 weeks of anti-TB treatment. If the deferral of HAART to 30 days after TB treatment did not increase mortality and can also reduce the incidence of IRIS, it would offer better flexibility for clinical judgment. This timing has not been discussed in previous RCTs. This finding is distinct from, yet complementary to, the results of previous RCTs and need more research to prove this hypothesis.

Our results showed that HIV/HCV co-infected patients had higher chance to have HAART withheld during TB treatment. Anti-TB drug-induced hepatotoxicity is the most common physician-determined reason to defer HAART initiation [34]. The median time to the development of hepatotoxicity is 14 days after TB treatment, thus the initiation of HAART would also be delayed or suspended because of abnormal liver function [35,36]. The rate of anti-TB drug-induced hepatotoxicity is high in Taiwan [37,38], and is even higher in HIV/HBV or HIV/HCV co-infected patients [36,39,40]. Our study found high prevalence of HCV co-infection in HIV-infected individuals, thus increased frequency of liver function monitoring before HAART initiation is necessary. Aggressive latent tuberculosis infection treatment or HCV related treatment in patients with HIV/HCV co-infection might be another option to solve the problem.

There are some limitations in our analysis. First, this is a retrospective study and the reason for a clinicians’ judgment as to the timing of HAART was not always clearly recorded. Second, this is a nationwide study and the data was collected from several hospitals. There were approximately 40 designated hospitals providing free HIV-related medical care in Taiwan, the majority of which were tertiary medical centers of excellent quality [20]. Around 82% of our patients were treated in medical centers and the mortality rate was 22%; this is not statistically different from patients who were treated in regional hospitals, where the mortality rate was 14%. Thus the quality of medical care did not show significant difference. Third, the case number was small and the distribution was uneven in our study, despite the study was based on Taiwan’s national HIV and TB surveillance dataset and can therefore be considered a population-based study for this area. In addition, the Taiwan government offers free HIV- and TB-related medical care through its national health insurance system, patients do not have to pay for treatment, leading to a higher percentage of individuals seeking HIV and TB related care. This situation, however, may not be representative of resource-constrained areas.

## Conclusion

In conclusion, the present study support that HAART initiated during TB treatment was associated with better one-year survival, though initiation within 60 days of TB treatment did not show statistical difference in survival than later initiation. Initiation of HAART within 30 days appeared to increase the risk of IRIS. Deferring HAART to 31–60 days of TB treatment might be optimal after considering the risks and benefits.

## Competing interests

There was no financial support for this study. On behalf of all authors, the corresponding author states that there is no conflict of interest.

## Authors’ contributions

CHY conceived of and designed this study and drafted the manuscript. HYC participated in the study design and analysis. KJC, JJT, YHL and SHC participated in the clinical data collection. KFW helped to link the datasets to identify those patients who matched the enrollment criteria. All authors were provided critical comments for revision and approved the final version of the manuscript.

## Pre-publication history

The pre-publication history for this paper can be accessed here:

http://www.biomedcentral.com/1471-2334/14/304/prepub

## Supplementary Material

Additional file 1: Table S1 Univariate analysis of factors influencing TB mortality and IRIS occurrence among HIV/TB coinfected infections*. **Table S2.** The relationship of mortality and initiation timing of HAART among HIV-PTB co-infected patients by CD4+ lymphocyte count > or ≤ 50 cells/mm3. **Table S3.** The relationship of IRIS occurrence and initiation timing of HAART among HIV-TB co-infected patients by CD4+ lymphocyte count > or ≤ 50 cells/mm3. Click here for file
