# Supplementary material for: The impact of HAART initiation timing on HIV-TB co-infected patients, a retrospective cohort study
Source: BMC Infect Dis. 2014 Jun 4;14:304. doi: 10.1186/1471-2334-14-304 (PMC4058447; doi:10.1186/1471-2334-14-304)
Supplement: Additional file 1: Table S1 — Univariate analysis of factors influencing TB mortality and IRIS occurrence among HIV/TB coinfected infections*. Table S2. The relationship of mortality and initiation timing of HAART among HIV-PTB co-infected patients by CD4+ lymphocyte count > or ≤ 50 cells/mm3. Table S3. The relationship of IRIS occurrence and initiation timing of HAART among HIV-TB co-infected patients by CD4+ lymphocyte count > or ≤ 50 cells/mm3. [file 1471-2334-14-304-S1.docx]

Additional file:

Supplementary Table 1. Univariate analysis of factors influencing TB mortality and IRIS occurrence among HIV/TB coinfected infections*.

|  | Death(n=52, 24%) | | IRIS(n=57, 25%)* | |
| --- | --- | --- | --- | --- |
|  | Hazard Ratio (95% CI) | *p* value | Hazard Ratio (95% CI) | *p* value |
| Age at TB diagnosis (per 5 years increase) | 1.14(1.03-1.27 | 0.01 | 0.95(0.84-1.07) | 0.36 |
| TB diagnose Year | 1.04(0.94-1.15) | 0.45 | 1.04(0.94-1.14) | 0.46 |
| Female | 1.44(0.52-4.00) | 0.48 | 2.09(0.76-5.79) | 0.16 |
| CD4 count at TB diagnosis (per 50 cells increase) | 0.94(0.83-1.06) | 0.29 | 0.95(0.81-1.11) | 0.50 |
| Localization of tuberculosis |  |  |  |  |
| Pulmonary | 1 |  | 1 |  |
| PTB+ extra-pulmonary | 1.19(0.66-2.12) | 0.56 | 1.82(0.97-3.40) | 0.06 |
| extra-pulmonary | 0.26(0.04-1.97) | 0.19 | 1.57(0.56-4.41) | 0.39 |
| Laboratory findings |  |  |  |  |
| Positive AFS smear | 1.23(0.63-2.39) | 0.55 | 1.02(0.55-1.90) | 0.94 |
| Positive MTB culture | 0.80(0.41-1.56) | 0.51 | 2.64(1.06-6.62) | 0.04 |
| HBV(+) | 1.15(0.48-2.72) | 0.75 | 0.58(0.26-1.29) | 0.18 |
| HCV(+) | 0.90(0.31-2.62) | 0.84 | 0.55(0.22-1.33) | 0.21 |
| IRIS | 0.26(0.10-0.65) | <0.01 |  |  |
| HAART( no HAART as reference) | 0.15(0.09-0.26) | <0.01 |  |  |
| HAART initiation timing during anti-TB therapy | | | | |
| No HAART | 1 |  |  |  |
| 0-15 days | 0.17(0.09-0.31) | <0.01 | 1 |  |
| 16-30 days | 0.14(0.05-0.36) | <0.01 | 0.96(0.50-1.84) | 0.94 |
| 31-60 days | -- |  | 0.24(0.06-0.98) | 0.04 |
| >60 days | 0.25(0.10-0.67) | <0.01 | 0.27(0.07-1.12) | 0.06 |

Abbreviation: HAART: highly active antiretroviral therapy; IRIS: Immune reconstitution inflammatory syndrome.

*Only enrolled cases with available CD4+ lymphocyte count and start HAART during TB treatment.

Supplementary Table 2.The relationship of mortality and initiation timing of HAART among HIV-PTB co-infected patients by CD4+ lymphocyte count > or ≤ 50 cells/mm^3^.

| **Cases with CD4** ≤**50 count(/mm^3^) at TB diagnosis** | | | | |  |
| --- | --- | --- | --- | --- | --- |
|  | Total*  (N=127) | Death  (N=32, 25%) | Hazard Ratio  (95% CI) | aHR(95% CI)^$^ | Adjusted HR^#^  (95% CI) |
| Age at TB diagnosis(per 5 years increase) | | | 1.17(1.02-1.33) | 1.19(1.03-1.37) | 1.25(1.03-1.51) |
| IRIS | 34 | 0 | -- | -- | -- |
| HAART initiation timing during anti-TB therapy | | | | | |
| No HAART | 14 | 13(92.9%) | 1 | 1 |  |
| 0-15 days | 68 | 13(19.1%) | 0.07(0.03-0.16) | 0.13 (0.06-0.30) | 1.78(0.46-6.92) |
| 16-30 days | 26 | 3(11.5%) | 0.04(0.01-0.15) | 0.06(0.02-0.22) | 0.79(0.15-4.32) |
| 31-60 days | 8 | 0(0%) | -- | -- | -- |
| >60 days | 11 | 3(27.3%) | 0.10(0.03-0.37) | 0.08(0.02-0.31) | 1 |
| **Cases with CD4 > 50 count(/mm^3^) at TB diagnosis** | | | | |  |
|  | Total*  (N=91) | Death  (N=20, 22%) | Hazard Ratio  (95% CI) | aHR(95% CI)^$^ | Adjusted HR^#^  (95% CI) |
| Age at TB diagnosis(per 5 years increase) | | | 1.10(0.92-1.35) | 1.11(0.92-1.35) | 1.00(0.76-1.33) |
| IRIS | 23 | 5(21.7%) | 0.92(0.33-2.52) | 2.12(0.56-7.99) | 1.91(0.51-7.22) |
| HAART initiation timing during anti-TB therapy | | | | | |
| No HAART | 22 | 10(45.5%) | 1 | 1 |  |
| 0-15 days | 42 | 6(14.3%) | 0.24(0.09-0.65) | 0.18(0.05-0.61) | 0.50(0.09-2.73) |
| 16-30 days | 8 | 2(25%) | 0.43(0.10-1.98) | 0.27(0.05-1.59) | 0.84(0.11-6.65) |
| 31-60 days | 11 | 0(0%) | -- | -- | -- |
| >60 days | 8 | 2(25.0%) | 0.40(0.09-1.81) | 0.30(0.06-1.48) | 1 |
| Abbreviation: HAART: highly active antiretroviral therapy; IRIS: Immune reconstitution inflammatory syndrome.  * Only enrolled cases with available CD4+ lymphocyte count. HBV co-infection, HCV co-infection and TB location were analyzed initially but the p value was greater than 0.15 and was not included for multivariate analysis and not shown in the table.  $ Adjusted for age at TB diagnosis, IRIS and HAART initiation timing(use no HAART as reference).  # Excluded cases who didn’t start HAART during TB treatment and adjusted for age at TB diagnosis, IRIS and HAART initiation timing (use after 60 day as reference). | | | | | |

Supplementary Table 3. The relationship of IRIS occurrence and initiation timing of HAART among HIV-TB co-infected patients by CD4+ lymphocyte count > or ≤ 50 cells/mm^3^.

| **Cases with CD4** ≤**50 count(/mm^3^) at TB diagnosis** | | | | | |
| --- | --- | --- | --- | --- | --- |
|  | | Total*  (n=113) | IRIS  (N=34, 30%) | Hazard Ratio  (95% CI) | Adjusted HR^#^  (95% CI) |
| Age at TB diagnosis (per 5 year increase) | |  |  | 1.00(0.86-1.16) | 1.03(0.88-1.21) |
| HAART initiation timing during anti-TB therapy | | | | | |
| 0-15 days | | 68 | 26(28.2%) | 1 | 1 |
| 16-30 days | | 26 | 7(26.9%) | 0.65(0.28-1.51) | 0.66(0.29-1.52) |
| 31-60 days | | 8 | 0(0%) | -- | -- |
| >60 days | | 11 | 1(9.1%) | 0.24(0.03-1.75) | 0.23(0.03-1.70) |
|  | |  |  |  |  |
| **Cases with CD4 > 50 count(/mm^3^) at TB diagnosis** | | | | | |
|  | | Total*  (n=69) | IRIS  (N=23, 33%) | Hazard Ratio  (95% CI) | Adjusted HR#  (95% CI) |
| Age at TB diagnosis (per 5 year increase) | |  |  | 0.88(0.72-1.07) | 0.87(0.71-1.07) |
| HAART initiation timing during anti-TB therapy | | | | | |
| 0-15 days | | 42 | 15(35.7%) | 1 | 1 |
| 16-30 days | | 8 | 5(62.5%) | 2.72(0.98-7.53) | 3.09(1.09-8.75) |
| 31-60 days | | 11 | 2(18.2%) | 0.43(0.10-1.87) | 0.46(0.11-2.03) |
| >60 days | | 8 | 1(12.5%) | 0.29(0.04-2.16) | 0.32(0.04-2.40) |
| Abbreviation: HAART: highly active antiretroviral therapy; IRIS: Immune reconstitution inflammatory syndrome.  * Only enrolled cases with available CD4+ lymphocyte count. HBV co-infection, HCV co-infection and TB location were analyzed initially but the p value was greater than 0.1 and was not included for multivariate analysis and not shown in the table.  $ Adjusted for age at TB diagnosis, IRIS and HAART initiation timing (use no HAART as reference).  # Excluded cases who didn’t start HAART during TB treatment and adjusted for age at TB diagnosis, IRIS and HAART initiation timing (use after 60 day as reference). | | | | | |
